# Dibromido(di-2-pyridyl­amine-κ^2^ *N* ^2^,*N* ^2′^)platinum(II)

**DOI:** 10.1107/S1600536812011300

**Published:** 2012-03-21

**Authors:** Kwang Ha

**Affiliations:** a School of Applied Chemical Engineering, Research Institute of Catalysis, Chonnam National University, Gwangju 500-757, Republic of Korea

## Abstract

The Pt^II^ ion in the title complex, [PtBr_2_(C_10_H_9_N_3_)], is four-coordinated in an essentially square-planar environment by two N atoms from a chelating di-2-pyridyl­amine (dpa) ligand and two Br^−^ anions. The dpa ligand is not planar, with the dihedral angle between the pyridine rings being 40.8 (2)°. The complex mol­ecules are stacked in columns along [001] through π–π inter­actions between the pyridine rings [centroid–centroid distances = 3.437 (3) and 3.520 (3) Å]. Inter­molecular N—H⋯Br hydrogen bonds connect the mol­ecules into chains running along [010]. Intra­molecular C—H⋯Br interactions are also observed.

## Related literature

For the structure of a related chlorido Pt^II^ complex [PtCl_2_(dpa)], see: Li & Liu (2004 ▶); Tu *et al.* (2004 ▶); Zhang *et al.* (2006 ▶).
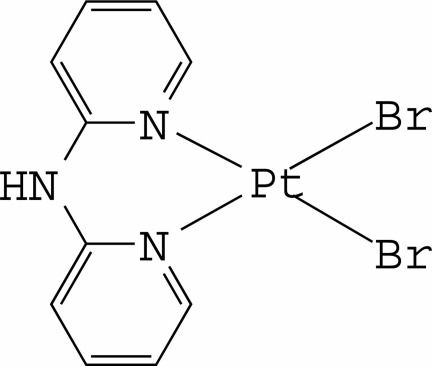


## Experimental

### 

#### Crystal data

[PtBr_2_(C_10_H_9_N_3_)]
*M* *_r_* = 526.08
Orthorhombic, 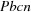

*a* = 12.900 (2) Å
*b* = 14.004 (3) Å
*c* = 13.440 (3) Å
*V* = 2428.0 (8) Å^3^
*Z* = 8
Mo *K*α radiation
μ = 18.12 mm^−1^
*T* = 200 K
0.27 × 0.25 × 0.24 mm

#### Data collection

Bruker SMART 1000 CCD diffractometer
Absorption correction: multi-scan (*SADABS*; Bruker, 2001 ▶) *T*_min_ = 0.700, *T*_max_ = 1.000
15922 measured reflections
2973 independent reflections
2424 reflections with *I* > 2σ(*I*)
*R*_int_ = 0.049

#### Refinement

*R*[*F* ^2^ > 2σ(*F* ^2^)] = 0.029
*wR*(*F* ^2^) = 0.079
*S* = 1.10
2973 reflections
145 parameters
H-atom parameters constrained
Δρ_max_ = 1.41 e Å^−3^
Δρ_min_ = −2.22 e Å^−3^

### 

Data collection: *SMART* (Bruker, 2007 ▶); cell refinement: *SAINT* (Bruker, 2007 ▶); data reduction: *SAINT*; program(s) used to solve structure: *SHELXS97* (Sheldrick, 2008 ▶); program(s) used to refine structure: *SHELXL97* (Sheldrick, 2008 ▶); molecular graphics: *ORTEP-3* (Farrugia, 1997 ▶) and *PLATON* (Spek, 2009 ▶); software used to prepare material for publication: *SHELXL97*.

## Supplementary Material

Crystal structure: contains datablock(s) global. DOI: 10.1107/S1600536812011300/hy2523sup1.cif

Additional supplementary materials:  crystallographic information; 3D view; checkCIF report

## Figures and Tables

**Table 1:** Selected bond lengths (Å)

Pt1—N1	2.031 (5)
Pt1—N3	2.026 (5)
Pt1—Br1	2.4198 (7)
Pt1—Br2	2.4282 (7)

**Table 2:** Hydrogen-bond geometry (Å, °)

*D*—H⋯*A*	*D*—H	H⋯*A*	*D*⋯*A*	*D*—H⋯*A*
N2—H2*N*⋯Br1^i^	0.92	2.59	3.510 (4)	178
C1—H1⋯Br1	0.95	2.89	3.288 (6)	107
C10—H10⋯Br2	0.95	2.84	3.241 (6)	106

Symmetry code: (i) 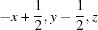.

**Fig. 1.**
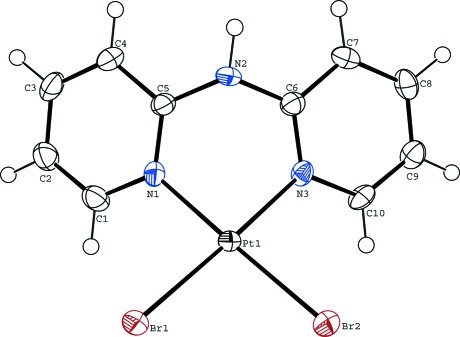
Molecular structure of the title complex, with displacement ellipsoids drawn at the 50% probability level.

**Fig. 2.**
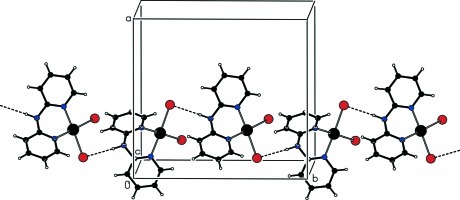
A partial view of the unit-cell contents of the title complex. Intermolecular N—H···Br hydrogen bonds are drawn as dashed lines.

## supplementary crystallographic information

### Comment

The title complex, [PtBr_2_(dpa)] (dpa = di-2-pyridylamine), crystallizes in the orthorhombic space group *Pbcn*, whereas the analogous chlorido Pt^II^ complex [PtCl_2_(dpa)] crystallizes in the monoclinic space group *P*2_1_/*n* (Li & Liu, 2004; Tu *et al.*, 2004; Zhang *et al.*, 2006).

The Pt^II^ ion is four-coordinated in an essentially square-planar environment by two N atoms from a chelating dpa ligand and two Br^-^ anions (Fig. 1). The Pt—N and Pt—Br bond lengths are nearly equivalent, respectively (Table 1). The dpa ligand is not planar. The dihedral angle between the least-squares planes of the pyridine rings is 40.8 (2)°. In the crystal, the complex molecules are stacked in columns along [001] through π–π interactions between the pyridine rings [centroid–centroid distances = 3.437 (3) and 3.520 (3) Å]. Intermolecular N—H···Br hydrogen bonds connect the molecules into chains running along [010] (Fig. 2, Table 2). Intramolecular C—H···Br hydrogen bonds are also observed.

### Experimental

To a solution of K_2_PtBr_4_ (0.2326 g, 0.392 mmol) in H_2_O (20 ml) and MeOH (10 ml) was added di-2-pyridylamine (0.0722 g, 0.422 mmol) and stirred for 7 h at room temperature. The formed precipitate was separated by filtration, washed with H_2_O and acetone and dried at 50°C to give a yellow powder (0.1502 g). Crystals suitable for X-ray analysis were obtained by slow evaporation from an acetone solution.

### Refinement

C-bound H atoms were positioned geometrically and allowed to ride on their respective parent atoms [C—H = 0.95 Å and *U*_iso_(H) = 1.2*U*_eq_(C)]. N-bound H atom was located from a difference Fourier map and allowed to ride on its parent atom in the final cycles of refinement, with N—H = 0.92 Å and *U*_iso_(H) = 1.5*U*_eq_(N). The highest peak (1.41 e Å^-3^) and the deepest hole (-2.22 e Å^-3^) in the difference Fourier map are located 0.73 and 0.79 Å from Pt1 atom, respectively.

### Crystal data

**Table:**

[PtBr_2_(C_10_H_9_N_3_)]	*F*(000) = 1904
*M**_r_* = 526.08	*D*_x_ = 2.878 Mg m^−^^3^
Orthorhombic, *P**b**c**n*	Mo *K*α radiation, λ = 0.71073 Å
Hall symbol: -P 2n 2ab	Cell parameters from 8242 reflections
*a* = 12.900 (2) Å	θ = 2.6–28.3°
*b* = 14.004 (3) Å	µ = 18.12 mm^−^^1^
*c* = 13.440 (3) Å	*T* = 200 K
*V* = 2428.0 (8) Å^3^	Block, yellow
*Z* = 8	0.27 × 0.25 × 0.24 mm

### Data collection

**Table:**

Bruker SMART 1000 CCD diffractometer	2973 independent reflections
Radiation source: fine-focus sealed tube	2424 reflections with *I* > 2σ(*I*)
Graphite monochromator	*R*_int_ = 0.049
φ and ω scans	θ_max_ = 28.3°, θ_min_ = 2.2°
Absorption correction: multi-scan (*SADABS*; Bruker, 2001)	*h* = −17→17
*T*_min_ = 0.700, *T*_max_ = 1.000	*k* = −18→9
15922 measured reflections	*l* = −17→17

### Refinement

**Table:**

Refinement on *F*^2^	Primary atom site location: structure-invariant direct methods
Least-squares matrix: full	Secondary atom site location: difference Fourier map
*R*[*F*^2^ > 2σ(*F*^2^)] = 0.029	Hydrogen site location: inferred from neighbouring sites
*wR*(*F*^2^) = 0.079	H-atom parameters constrained
*S* = 1.10	*w* = 1/[σ^2^(*F*_o_^2^) + (0.0344*P*)^2^ + 3.6402*P*] where *P* = (*F*_o_^2^ + 2*F*_c_^2^)/3
2973 reflections	(Δ/σ)_max_ = 0.001
145 parameters	Δρ_max_ = 1.41 e Å^−^^3^
0 restraints	Δρ_min_ = −2.22 e Å^−^^3^

### Special details

**Table:**

Geometry. All e.s.d.'s (except the e.s.d. in the dihedral angle between two l.s. planes) are estimated using the full covariance matrix. The cell e.s.d.'s are taken into account individually in the estimation of e.s.d.'s in distances, angles and torsion angles; correlations between e.s.d.'s in cell parameters are only used when they are defined by crystal symmetry. An approximate (isotropic) treatment of cell e.s.d.'s is used for estimating e.s.d.'s involving l.s. planes.
Refinement. Refinement of *F*^2^ against ALL reflections. The weighted *R*-factor *wR* and goodness of fit *S* are based on *F*^2^, conventional *R*-factors *R* are based on *F*, with *F* set to zero for negative *F*^2^. The threshold expression of *F*^2^ > σ(*F*^2^) is used only for calculating *R*-factors(gt) *etc*. and is not relevant to the choice of reflections for refinement. *R*-factors based on *F*^2^ are statistically about twice as large as those based on *F*, and *R*- factors based on ALL data will be even larger.

### Fractional atomic coordinates and isotropic or equivalent isotropic displacement parameters (Å^2^)

**Table:**

	*x*	*y*	*z*	*U*_iso_*/*U*_eq_	
Pt1	0.239801 (16)	0.136089 (17)	0.383005 (17)	0.01923 (9)	
Br1	0.41607 (4)	0.19255 (5)	0.40120 (5)	0.02934 (16)	
Br2	0.18330 (4)	0.25482 (4)	0.50297 (5)	0.03000 (16)	
N1	0.2859 (3)	0.0336 (4)	0.2856 (3)	0.0225 (10)	
N2	0.1654 (3)	−0.0719 (3)	0.3584 (4)	0.0234 (10)	
H2N	0.1425	−0.1331	0.3701	0.035*	
N3	0.0943 (3)	0.0824 (4)	0.3727 (3)	0.0212 (10)	
C1	0.3579 (4)	0.0513 (5)	0.2139 (4)	0.0290 (14)	
H1	0.3817	0.1149	0.2048	0.035*	
C2	0.3966 (5)	−0.0185 (5)	0.1551 (5)	0.0318 (14)	
H2	0.4464	−0.0038	0.1053	0.038*	
C3	0.3631 (5)	−0.1113 (5)	0.1683 (5)	0.0311 (15)	
H3	0.3913	−0.1615	0.1291	0.037*	
C4	0.2886 (5)	−0.1303 (4)	0.2386 (5)	0.0286 (13)	
H4	0.2652	−0.1937	0.2496	0.034*	
C5	0.2478 (4)	−0.0546 (4)	0.2936 (4)	0.0223 (12)	
C6	0.0806 (4)	−0.0123 (4)	0.3698 (4)	0.0226 (12)	
C7	−0.0175 (5)	−0.0530 (5)	0.3824 (4)	0.0240 (12)	
H7	−0.0258	−0.1205	0.3824	0.029*	
C8	−0.1017 (4)	0.0057 (5)	0.3946 (4)	0.0274 (14)	
H8	−0.1684	−0.0205	0.4071	0.033*	
C9	−0.0884 (5)	0.1041 (5)	0.3886 (4)	0.0275 (14)	
H9	−0.1464	0.1458	0.3924	0.033*	
C10	0.0089 (5)	0.1399 (4)	0.3773 (4)	0.0243 (13)	
H10	0.0178	0.2071	0.3724	0.029*	

### Atomic displacement parameters (Å^2^)

**Table:**

	*U*^11^	*U*^22^	*U*^33^	*U*^12^	*U*^13^	*U*^23^
Pt1	0.01844 (13)	0.01519 (14)	0.02405 (15)	−0.00108 (8)	0.00116 (8)	−0.00043 (8)
Br1	0.0201 (3)	0.0234 (3)	0.0445 (4)	−0.0034 (2)	0.0014 (2)	−0.0063 (3)
Br2	0.0264 (3)	0.0252 (3)	0.0384 (4)	−0.0022 (2)	0.0051 (2)	−0.0104 (3)
N1	0.018 (2)	0.023 (3)	0.026 (3)	0.002 (2)	0.0022 (19)	−0.002 (2)
N2	0.022 (2)	0.015 (2)	0.033 (3)	−0.0045 (19)	0.002 (2)	0.001 (2)
N3	0.016 (2)	0.023 (3)	0.024 (2)	0.0040 (19)	−0.0032 (18)	−0.003 (2)
C1	0.026 (3)	0.030 (4)	0.031 (3)	−0.007 (3)	0.000 (2)	−0.002 (3)
C2	0.028 (3)	0.036 (4)	0.032 (3)	−0.005 (3)	0.007 (3)	−0.005 (3)
C3	0.031 (3)	0.028 (4)	0.034 (4)	0.009 (3)	0.003 (3)	−0.009 (3)
C4	0.030 (3)	0.018 (3)	0.037 (4)	0.004 (2)	0.001 (3)	0.000 (3)
C5	0.021 (3)	0.018 (3)	0.029 (3)	0.001 (2)	−0.002 (2)	−0.001 (2)
C6	0.026 (3)	0.021 (3)	0.022 (3)	0.000 (2)	0.001 (2)	0.000 (2)
C7	0.030 (3)	0.022 (3)	0.020 (3)	−0.008 (2)	−0.002 (2)	0.002 (2)
C8	0.015 (2)	0.040 (4)	0.027 (3)	−0.004 (3)	−0.003 (2)	−0.002 (3)
C9	0.022 (3)	0.031 (4)	0.030 (3)	0.004 (3)	−0.005 (2)	−0.006 (3)
C10	0.031 (3)	0.018 (3)	0.025 (3)	0.010 (2)	−0.003 (2)	0.003 (2)

### Geometric parameters (Å, º)

**Table:**

Pt1—N1	2.031 (5)	C2—H2	0.9500
Pt1—N3	2.026 (5)	C3—C4	1.375 (9)
Pt1—Br1	2.4198 (7)	C3—H3	0.9500
Pt1—Br2	2.4282 (7)	C4—C5	1.394 (8)
N1—C5	1.334 (8)	C4—H4	0.9500
N1—C1	1.362 (7)	C6—C7	1.399 (8)
N2—C6	1.384 (7)	C7—C8	1.372 (9)
N2—C5	1.396 (7)	C7—H7	0.9500
N2—H2N	0.9200	C8—C9	1.392 (9)
N3—C6	1.338 (8)	C8—H8	0.9500
N3—C10	1.366 (7)	C9—C10	1.360 (9)
C1—C2	1.352 (9)	C9—H9	0.9500
C1—H1	0.9500	C10—H10	0.9500
C2—C3	1.382 (9)		
			
N3—Pt1—N1	87.97 (19)	C2—C3—H3	120.4
N3—Pt1—Br1	176.75 (14)	C3—C4—C5	118.8 (6)
N1—Pt1—Br1	91.20 (13)	C3—C4—H4	120.6
N3—Pt1—Br2	91.24 (14)	C5—C4—H4	120.6
N1—Pt1—Br2	178.26 (14)	N1—C5—C4	121.5 (5)
Br1—Pt1—Br2	89.50 (2)	N1—C5—N2	119.4 (5)
C5—N1—C1	118.4 (5)	C4—C5—N2	119.1 (5)
C5—N1—Pt1	119.7 (4)	N3—C6—N2	119.7 (5)
C1—N1—Pt1	121.8 (4)	N3—C6—C7	121.4 (5)
C6—N2—C5	124.6 (5)	N2—C6—C7	118.8 (5)
C6—N2—H2N	106.9	C8—C7—C6	119.1 (6)
C5—N2—H2N	120.8	C8—C7—H7	120.5
C6—N3—C10	118.6 (5)	C6—C7—H7	120.5
C6—N3—Pt1	119.5 (4)	C7—C8—C9	119.3 (6)
C10—N3—Pt1	121.7 (4)	C7—C8—H8	120.4
C2—C1—N1	122.3 (6)	C9—C8—H8	120.4
C2—C1—H1	118.8	C10—C9—C8	119.0 (6)
N1—C1—H1	118.8	C10—C9—H9	120.5
C1—C2—C3	119.3 (6)	C8—C9—H9	120.5
C1—C2—H2	120.3	C9—C10—N3	122.2 (6)
C3—C2—H2	120.3	C9—C10—H10	118.9
C4—C3—C2	119.2 (6)	N3—C10—H10	118.9
C4—C3—H3	120.4		
			
N3—Pt1—N1—C5	−40.4 (4)	C3—C4—C5—N1	−6.0 (9)
Br1—Pt1—N1—C5	136.5 (4)	C3—C4—C5—N2	174.0 (6)
N3—Pt1—N1—C1	142.0 (5)	C6—N2—C5—N1	41.5 (8)
Br1—Pt1—N1—C1	−41.1 (4)	C6—N2—C5—C4	−138.5 (6)
N1—Pt1—N3—C6	40.4 (4)	C10—N3—C6—N2	175.6 (5)
Br2—Pt1—N3—C6	−138.0 (4)	Pt1—N3—C6—N2	−10.0 (7)
N1—Pt1—N3—C10	−145.4 (4)	C10—N3—C6—C7	−7.1 (8)
Br2—Pt1—N3—C10	36.1 (4)	Pt1—N3—C6—C7	167.3 (4)
C5—N1—C1—C2	−4.3 (9)	C5—N2—C6—N3	−41.5 (8)
Pt1—N1—C1—C2	173.3 (5)	C5—N2—C6—C7	141.2 (6)
N1—C1—C2—C3	−0.6 (10)	N3—C6—C7—C8	2.1 (8)
C1—C2—C3—C4	2.2 (10)	N2—C6—C7—C8	179.5 (5)
C2—C3—C4—C5	1.0 (10)	C6—C7—C8—C9	3.6 (8)
C1—N1—C5—C4	7.6 (8)	C7—C8—C9—C10	−4.3 (9)
Pt1—N1—C5—C4	−170.1 (4)	C8—C9—C10—N3	−0.7 (9)
C1—N1—C5—N2	−172.4 (5)	C6—N3—C10—C9	6.4 (8)
Pt1—N1—C5—N2	9.9 (7)	Pt1—N3—C10—C9	−167.8 (4)

### Hydrogen-bond geometry (Å, º)

**Table:**

*D*—H···*A*	*D*—H	H···*A*	*D*···*A*	*D*—H···*A*
N2—H2*N*···Br1^i^	0.92	2.59	3.510 (4)	178
C1—H1···Br1	0.95	2.89	3.288 (6)	107
C10—H10···Br2	0.95	2.84	3.241 (6)	106

Symmetry code: (i) −*x*+1/2, *y*−1/2, *z*.
